# CircECE1 promotes osteosarcoma progression through regulating RAB3D by sponging miR-588

**DOI:** 10.1186/s13018-023-04045-4

**Published:** 2023-08-09

**Authors:** Zhizhong Liang, Yuxia Shi, Zhe Guan

**Affiliations:** Department of Bone and Soft Tissue Oncology, Shanxi Province Cancer Hospital, Shanxi Hospital Affiliated to Cancer Hospital, Chinese Academy of Medical Sciences, Cancer Hospital Affiliated to Shanxi Medical University, No.3, Zhigong New Street, Xinghualing District, Taiyuan, 030013 China

**Keywords:** circECE1, Microrna-588, RAB3D, Member RAS oncogene family, Osteosarcoma

## Abstract

**Background:**

Circular RNAs (circRNAs) have been confirmed to be involved in cancer pathogenesis. However, the underlying mechanism of circRNA endothelin converting enzyme 1 (circECE1) in osteosarcoma (OS) development is still not understood.

**Methods:**

The expression levels of circECE1, microRNA-588 (miR-588) and RAB3D, member RAS oncogene family (RAB3D) were gauged by quantitative real-time polymerase chain reaction (qRT-PCR) and western blot. OS cell proliferation was assessed by cell counting kit-8 (CCK-8) assay and 5-ethynyl-2’-deoxyuridine (EdU) assay. OS cell apoptosis rate and metastasis were identified by flow cytometry and transwell assay. Dual-luciferase reporter analysis and RNA immunoprecipitation (RIP) assay were performed to confirm the interactions among circECE1, miR-588 and RAB3D. Xenograft tumor models were established to explore circECE1 function in vivo. Immunohistochemistry (IHC) assay was applied to analyze RAB3D level after circECE1 knockdown.

**Results:**

In OS, circECE1 expression was higher than that in normal chondroma tissues. High levels of circECE1 were positively linked to OS cell viability, proliferation, migration and invasion, and negatively linked to OS cell apoptosis rate. It was found that circECE1 was a miR-588 sponge, and miR-588 inhibitor abrogated the influence of si-circECE1 on OS cells. MiR-588 targeted RAB3D to further regulate the pathological process of OS. Moreover, silencing circECE1 blocked OS tumor growth in vivo.

**Conclusion:**

We elucidated the function of a novel circECE1/miR-588/RAB3D axis in OS progression.

## Introduction

Osteosarcoma (OS) is a malignant tumor in children and adolescents [1, 2]. Apart from its highly aggressive feature [3], current therapies remain limited and the prognosis is still poor [4]. To increase the survival outcome in patients with OS, it is particularly critical to identify therapeutic targets against OS.

Circular RNAs (circRNAs) with a covalently closed loop structure were first identified in 1976 [5]. CircRNAs have been reported to function as sponges of certain microRNAs (miRNAs) [6, 7]. Therefore, circRNAs are implicated in the development of various cancers, such as ovarian cancer [8], colorectal cancer [9], and OS [10]. Shen et al*.* found that circRNA endothelin converting enzyme 1 (circECE1) has high levels in OS tissues, and it promotes energy metabolism through association with MYC proto-oncogene, bHLH transcription factor (c-Myc) [11]. Nonetheless, the mechanisms underlying the implication of circECE1 (hsa_circ_0002402) in OS events have not been sufficiently elucidated.

MiRNAs are a highly conserved type of noncoding RNAs that are about 22 nucleotides [12]. Many miRNAs can be targeted by circRNAs, and circRNAs can function as miRNA sponges [13]. In orthopedic conditions, miRNAs have been proposed as diagnostic biomarkers and therapeutic targets [14, 15]. Interestingly, accumulating evidence has indicated the critical role of miRNAs in all aspects of OS [16]. MiR-372-3p is shown by Gao et al*.* to suppress OS progression by regulating mitogen-activated protein kinase 7 (MAPK7) [17]. Zheng et al*.* demonstrated that miR-337-3p contributes to OS cell proliferation, migration and invasion via regulation of zinc finger protein 652 (ZNF652) expression [18]. Meanwhile, miR-588 can act as a tumor suppressor and modulate the poor prognosis of OS patients [19].

Ras-related gtp-binding protein, alternative splice (Rab) GTPases are involved in transport processes, such as exocytic and endocytic membrane trafficking [20]. RAB3D, member RAS oncogene family (RAB3D) is one of the key members of the Rab GTPase family and has been extensively studied for its ability to regulate important biological processes in various cancers [20]. High expression of RAB3D is closely related to OS cell proliferation and metastasis [21, 22]. The specific role of RAB3D in OS progression has not been fully studied.

Hence, this study elucidated the exact role and molecular process of circECE1 in OS. Via bioinformatics, the putative circECE1/miR-588 and miR-588/RAB3D relationships were predicted, hinting a potential circECE1/miR-588/RAB3D regulatory axis in OS progression. Our data indicated that circECE1 expression was higher in OS samples than that in normal chondroma tissues. Mechanistically, circECE1 was a miR-588 sponge, and miR-588 targeted RAB3D in OS cells, demonstrating the crucial involvement of circECE1/miR-588/RAB3D axis in OS process. This study might provide an innovative opportunity for OS treatment.

## Materials and methods

### Tissue collection and cell culture

Written informed consent was obtained from OS patients in our study. 111 paired OS tissues and matched normal adjacent tissues were collected from Shanxi Province Cancer Hospital. This study followed the Ethics Committee of Shanxi Province Cancer Hospital and Helsinki Declaration. The association between circECE1 expression and the clinicopathologic features of osteosarcoma patients is shown in Table 1. The human osteoblast cell line (hFOB1.19) and OS cell lines (MG63 and U2OS) were acquired from the American Type Culture Collection Center (ATCC, Manassas, VA, USA). These cells were kept in a 5% CO_2_ incubator at 37 °C in DMEM medium (cat#12491015; Thermo Fisher Scientific, Waltham, MA, USA) including 10% FBS (cat#10099141; Thermo Fisher Scientific), 100 U/ml Penicillin (Thermo Fisher Scientific), and 100 µg/ml Streptomycin (Thermo Fisher Scientific).

**Table 1:** The association between circECE1 expression and the clinicopathologic features of osteosarcoma patients

	*N* = 111	circECE1 expression		*P* value
		Low (*N* = 55)	High (*n* = 56)	
Gender				0.1845
Female	52	22	30	
Male	59	33	26	
Age (years)				0.5700
≤ 60	51	27	24	
> 60	60	28	32	
TNM grade				0.0003*
I + II	53	36	17	
III	58	19	39	
Lymph node metastasis				0.0136*
Positive	58	22	36	
Negative	53	33	20	
Tumor size				0.0001*
≤ 4 cm	50	35	15	
> 4 cm	61	20	41	

^*^P < 0.05

*TNM* tumor-node-metastasis

### Cell transfection

GenePharma (Shanghai, China) synthesized small interfering RNA against circECE1 (si-circECE1: 5′-TGTACGTCGACATCGAGGAGG-3′; siRNA target sequence: 5′-CCTCCTCGATGTCGACGTACA-3′), miR-588 inhibitor, RAB3D overexpression vector (pcDNA-RAB3D), miR-588 mimic and their negative controls (si-NC, inhibitor NC, pcDNA and mimic NC). All OS cell transfections were conducted utilizing Lipofectamine 2000 (cat#11668019; Invitrogen, Carlsbad, CA, USA). GeneChem (Shanghai, China) synthesized short hairpin RNA against circECE1 (sh-circECE1) and sh-NC.

### Quantitative real-time polymerase chain reaction (qRT-PCR)

RNA was extracted from OS samples and cells according to the TRIzol kit (cat#15596018; Invitrogen). Reverse transcription (RT) was done using the Bio-Rad iScript kit (cat#1708890; Bio-Rad, Hercules, CA, USA) with random hexamers (for circECE1 and mRNA expression analysis) or the miRNA 1st Strand cDNA Synthesis Kit with stem-loop RT primer (for miR-497-5p expression analysis; cat#MR101-01/02; Vazyme, Nanjing, China). CircECE1, endothelin converting enzyme 1 (ECE1) mRNA, miR-588 and RAB3D mRNA levels were determined through iQSYBR Green SuperMix (cat#1725852; Bio-Rad) with 2^−ΔΔCt^ method on Real-Time PCR Detection System (Bio-Rad). Glyceraldehyde 3-phosphate dehydrogenase (GAPDH) and U6 were selected as internal controls. Primers used in qRT-PCR are listed in Table 2.

**Table 2:** Primer sequences used for PCR

Name		Primers for PCR (5′–3′)
hsa_circ_0002402	Forward	ACCTCTGGGAACACAACCAA
	Reverse	GCCGTTGGGGTATGCGTC
RAB3D	Forward	CGACGACCTTGGTTTCGAGT
	Reverse	GGTTCCAGGGACTCGTTCAT
miR-588	Forward	TCGGCAGGTTGGCCACAATGGGT
	Reverse	CTCAACTGGTGTCGTGGAG
GAPDH	Forward	GACAGTCAGCCGCATCTTCT
	Reverse	GCGCCCAATACGACCAAATC
U6	Forward	CTCGCTTCGGCAGCACA
	Reverse	AACGCTTCACGAATTTGCGT
ECE1	Forward	GAGGAGGACCTGGTGGACTC
	Reverse	CGCCAGAAGTACCACCAACA

### RNase R and actinomycin D (ActD) assays

CircECE1 structure was verified by RNase R and ActD assays. Briefly, 2 μg RNA was digested with 3 U/μg RNase R (cat#R0301; Geneseed, Guangzhou, China) for 30 min. In ActD assay, OS cells were treated with ActD (cat#SBR00013; Sigma-Aldrich, Louis, MO, USA) and collected at different time points. Finally, the RNA levels of circECE1 and linear ECE1 were estimated by qRT-PCR.

### Cell counting kit-8 (CCK-8) assay

The CCK-8 assay was applied to analyze OS cell proliferation. Transfected OS cells (3 × 10^3^) were cultivated into 96-well plates. After incubation for 0, 24, 48, 72 h, the growth medium in each well was replaced by fresh DMEM (100 μL) containing 10% FBS. CCK-8 reagent (10 μL, Dojindo, Tokyo, Japan) was added into each well, and followed by the incubation for 4 h. Lastly, the absorbance at 450 nm was gauged by a microplate reader (Bio-Rad).

### 5-Ethynyl-2’-deoxyuridine (EdU) assay

The BeyoClick™ EdU Cell Proliferation Kit (cat#C0071S; Beyotime, Shanghai, China) was applied for cell proliferation. Transfected OS cells were cultivated in 96-well dishes for 48 h, and the EdU (20 μM, 100 μL, Beyotime) medium was added to each well for 2 h. After that, 4% paraformaldehyde (Beyotime) was applied to fix OS cells, and DAPI (Beyotime) was applied for nucleus staining. Lastly, OS cell proliferation was observed by microscopy (50 μm, Olympus, Tokyo, Japan).

### Flow cytometry (FCM)

The Annexin V-FITC/PI apoptosis detection kit (cat#CA1020; Solarbio, Beijing, China) was applied to evaluate OS cell apoptosis. Transfected MG63 and U2OS cells (3 × 10^5^) were cultivated for 48 h and then cleaned with PBS. The cells were resuspended in a mixture of binding buffer (90 μL), Annexin V-FITC (5 μL) and PI (5 μL) at 25 °C for 20 min. Results were finally monitored by FCM (Agilent, Beijing, China).

### Western blot

Proteins from tissue samples and OS cells were obtained by utilizing RIPA buffer solution (Beyotime). The extracted proteins were separated by SDS-PAGE. Afterwards, protein bands were transferred onto PVDF membranes (Millipore, Billerica, MA, USA). The primary and secondary antibodies were purchased from Abcam (Cambridge, MA, USA). The membranes were incubated with primary antibodies overnight at 4 °C, including anti-B-cell lymphoma 2 (Bcl-2, cat#ab182858, 1:5000), anti-Bcl2-associated X (Bax, cat#ab35203, 1:8000), anti-RAB3D (cat#ab133301, 1:3000), and anti-GAPDH (cat#ab8245, 1:20000). Next, the membranes were incubated with the secondary antibody (cat#ab6728, 1:2000) at room temperature for 1 h. Blots were visualized through immunoblotting chemiluminescence reagent ECL (Auragene, Changsha, China) and observed by ImageQuant LAS 500 (GE Healthcare, Piscataway, NJ, USA).

### Transwell assay

OS cells were collected 48 h after transfection. For invasion assay, OS cells (1 × 10^5^) were placed into the upper chamber pre-coated with matrigel (BD Bioscience, Bedford, MA, USA). The lower chamber was supplemented with DMEM plus 10% FBS. Afterwards, cells at the lower membrane were fixed with 4% paraformaldehyde (Beyotime) and colored with 0.1% crystal violet (Beyotime). Unlike invasion assay, the migration assay was implemented in the upper chamber without matrigel. Images were captured using a microscope (Leica, Shanghai, China).

### Wound-healing assay

Transfected OS cells in 6-well dishes were allowed to reach about 80% confluence. A linear wound was made by the 200 μL pipette tip. After 24 h of incubation, images were observed under microscopy (Leica).

### Dual-luciferase reporter assay

Starbase 3.0 (https://starbase.sysu.edu.cn/) was used to predict these binding sites about circECE1 to miR-588 and miR-588 to RAB3D. We inserted the fragments of circECE1 and RAB3D 3’UTR harboring wild or mutant target sequences into the pGL3 vector (Promega, Madison, WI, USA) to generate circECE1 wt, circECE1 mut, RAB3D 3’UTR wt and RAB3D 3’UTR mutant, respectively. The respective reporter vector and mimic NC or miR-588 mimic were then co-transfected into 293 T cells. Dual-Luciferase Reporter Assay kit (Hanbio, Shanghai, China) was employed for assessment of luciferase activity.

### RNA immunoprecipitation (RIP) assay

RIP assays were implemented utilizing the EZ-Magna RIP RNA-Binding Protein Immunoprecipitation Kit (Millipore). OS cells were homogenized in RIP buffer. After addition of anti-Ago2 or IgG antibody (Bioss, Beijing, China) and agarose beads, an 8-h incubation was allowed at 4 °C. After that, beads were digested using proteinase K (Solarbio), and RNA purification was carried out. Finally, the enrichment levels of circECE1 and miR-588 were examined by qRT-PCR.

### Experimental animals

A total of 12 male BALB/c nude mice (16–20 g; 5-week-old) were obtained from Vital River Laboratory Animal Technology (Beijing, China). MG63 cells (2 × 10^5^) stably transfected with sh-NC or sh-circECE1 lentivirus (RiboBio, Guangzhou, China) were subcutaneously injected into nude mice (6 mice/group). Tumor volume was weekly calculated in accordance with the formula: volume (mm^3^) = width^2^ × length/2. On week 4 after injection, tumors were removed from mice and weighed. Our project was ratified by the Animal Care and Use Committee of Shanxi Province Cancer Hospital.

### Immunohistochemistry (IHC)

OS tissues were washed with PBS and fixed with 4% paraformaldehyde (Beyotime), and paraffin-embedded sections were prepared. Anti-Ki-67 antibody (cat#ab15580, 1:1000, Abcam) and a secondary antibody (cat#ab150113, 1:1000, Abcam) were used as described by the producer, and the Ki67-positive cells were counted in the visualized fields.

### Statistical analysis

Unless otherwise indicated, all experiments were performed with at least 3 biological replicates and 3 technical replicates. Data processing was done using SPSS19.0 (IBM, Chicago, IL, USA). Student’s *t*-test or one-way analysis of variance (ANOVA) was employed to compare differences. *P* < 0.05 meant statistically significant difference.

## Results

### CircECE1 is highly expressed in OS tissues and cells

**Fig. 1:**
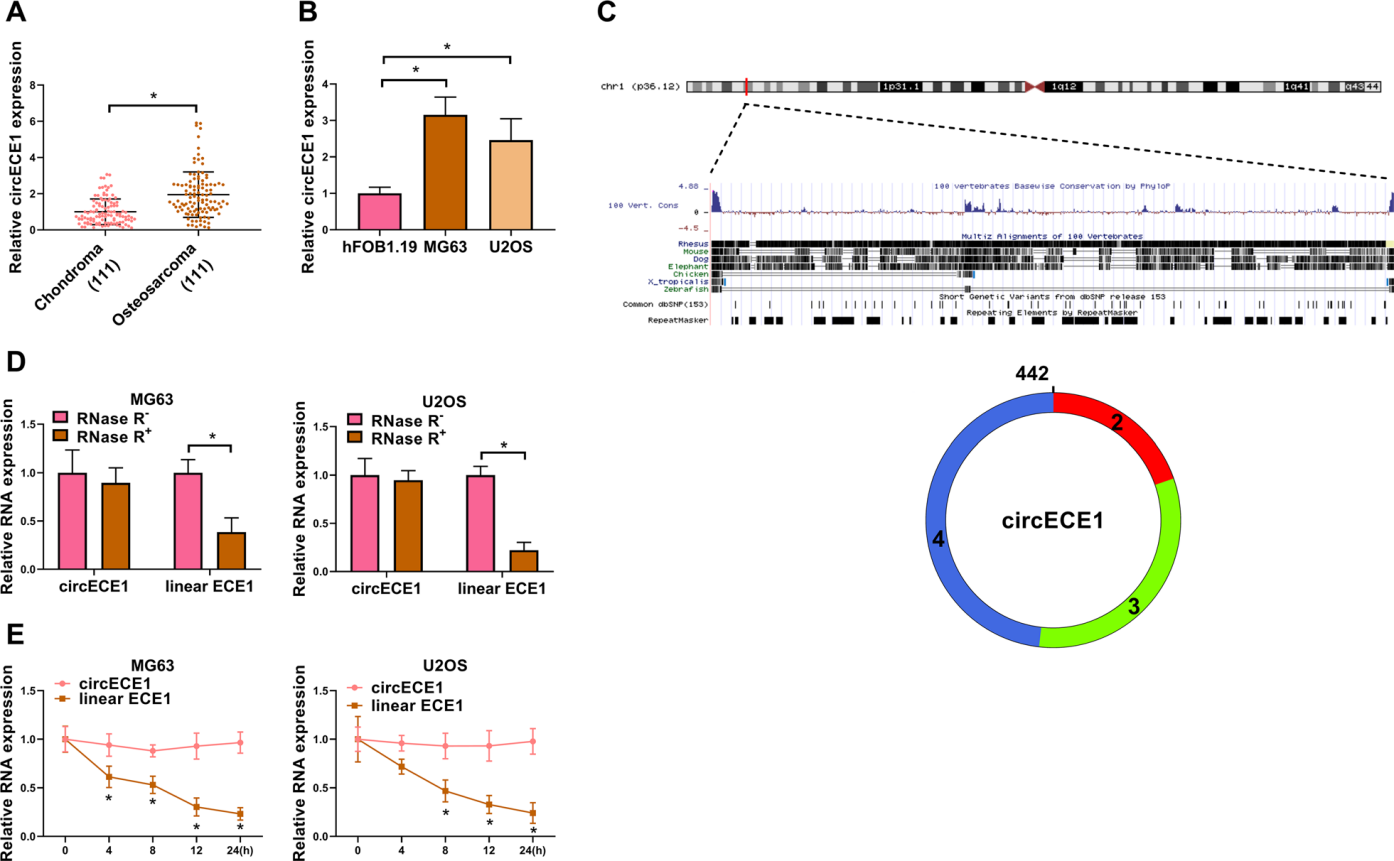
Upregulation of circECE1 in OS tissues and cells. **A** The expression levels of circECE1 in OS tissues (*N* = 111) and adjacent normal tissues (*N* = 111) were detected by qRT-PCR. GADPH was used as the internal control. This experiment was performed with 111 independent biological repeats. *P < 0.05. **B** The expression levels of circECE1 in OS cells (MG63 and U2OS) and hFOB1.19 cells were analyzed by qRT-PCR, with GAPDH as a reference gene. This experiment was performed with 3 independent biological repeats × 3 technique repeats. *P < 0.05. **C** The circular structure of circECE1, si-circECE1 sequence and its targeting location. **D** Total RNA of OS cells (MG63 and U2OS) was digested by RNase R. The levels of circECE1 and linear ECE1 were detected. The assay was performed with 3 independent biological repeats × 3 technique repeats. *P < 0.05. **E** OS (MG63 and U2OS) cells were treated with ActD at different time points. The expression levels of circECE1 and linear ECE1 by qRT-PCR, with GAPDH as a reference gene. This experiment was performed with 3 independent biological repeats × 3 technique repeats. *P < 0.05

We used qRT-PCR to examine the expression of circECE1 in 111 chondroma and 111 OS samples and found circECE1 expression was higher in OS tissues and cells (MG63 and U2OS) than that in the corresponding counterparts (Fig. 1A and B). In addition, we found that the high expression of circECE1 was closely associated with advanced TNM stage, positive lymph node metastasis, and large tumor size (Table 1). As shown in Fig. 1C, the structure of circECE1 was shown, which has a sequence length of 442 nt and is formed by exons 2, 3 and 4 of ECE1 pre-mRNA (Fig. 1C). To investigate the stability of circECE1, RNase R and ActD assays were performed. RNase R assays manifested that circECE1 was resistant to RNase R, while ECE1 mRNA was not resistant to RNase R (Fig. 1D). After ActD treatment, circECE1 level did not significantly change, but linear ECE1 RNA level sharply decreased, proving the circular structure of circECE1 (Fig. 1E). These data indicated that circECE1 might be linked to OS progression.

### Silencing of circECE1 curbs cell viability, migration and invasion in OS cells

**Fig. 2:**
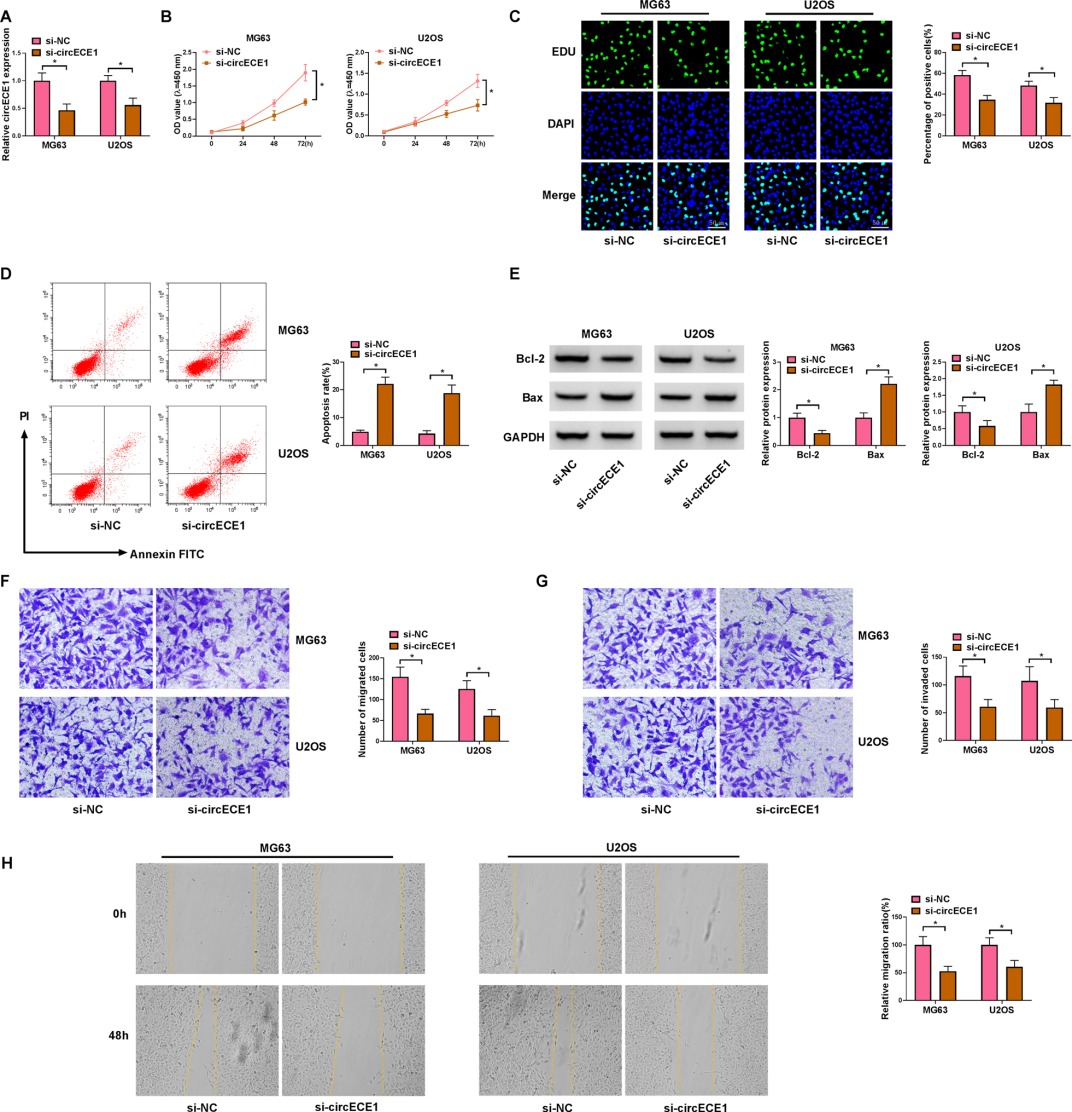
The effect of circECE1 knockdown in OS cells. **A**–**H** OS cells (MG63 and U2OS) were transiently transfected with si-circECE1 or si-NC. **A** The knockdown efficiency of si-circECE1 was observed by qRT-PCR, with GAPDH as a reference gene. **B** CCK-8 assay was adopted to detect OS cell viability. **C** EdU assay detected OS cell proliferation. **D** The apoptosis rate of OS cells was observed through FCM after transfection of si-NC or si-circECE1. **E** The protein levels of Bcl-2 and Bax were examined by western blot. **F** and **G** Transwell assays revealed the cell migration and invasion after transfection. **H** The migration ratio of OS cells was detected by wound-healing assay after si-circECE1 transfection. These experiments **A**, **B**, **C**, **D**, **E**, **F**, **G** and **H** were performed with 3 independent biological repeats × 3 technique repeats. *P < 0.05

To observe the function of circECE1, we transfected si-circECE1 into MG63 and U2OS cells. CircECE1 expression was suppressed by si-circECE1 transfection (Fig. 2A). Cell growth was further assessed using CCK-8 and EDU analyses, which indicated that silencing circECE1 impaired OS cell viability and proliferation (Fig. 2B and C). Silencing circECE1 augmented OS cell apoptosis (Fig. 2D). Western blot revealed that Bcl-2 expression was constrained and Bax expression was improved in OS cells with si-circECE1 introduction (Fig. 2E). Moreover, the invasion and migration abilities of OS cells (MG63 and U2OS) were prominently inhibited by circECE1 knockdown (Fig. 2F and G). Similarly, wound-healing assays presented that the migration ratio of OS cells was notably curbed by si-circECE1 (Fig. 2H). Taken together, circECE1 knockdown restricted the proliferation and metastasis and facilitated apoptosis in OS cells.

### CircECE1 acts as a miR-588 sponge in OS cells

**Fig. 3:**
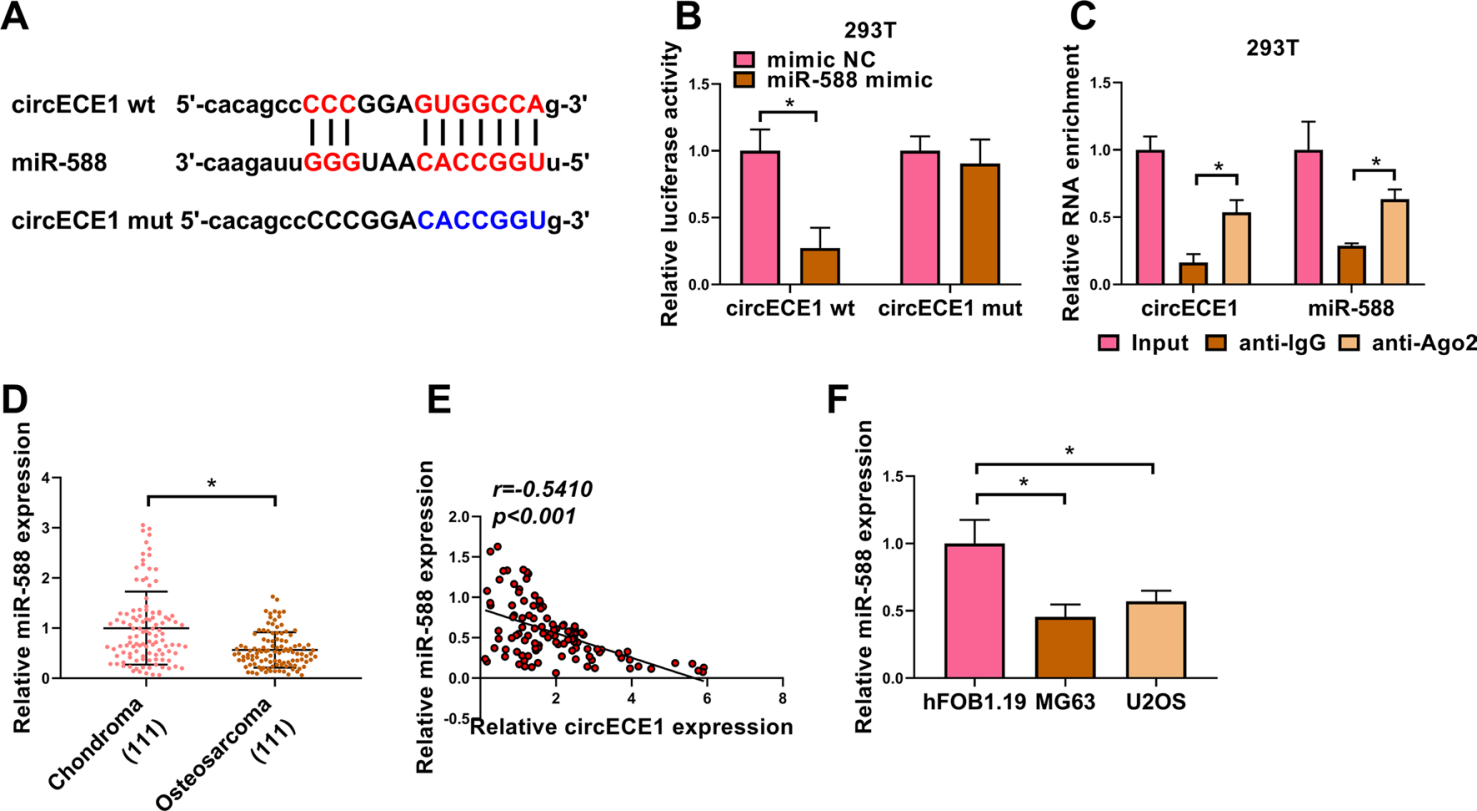
CircECE1 interacts with miR-588 in OS cells. **A** The binding sites between circECE1 and miR-588 were estimated by Starbase 3.0 software. **B** Dual-luciferase reporter assay was used to probe the relationship between circECE1 and miR-588. 293 T cells were co-transfected with circECE1 wt or circECE1 mut and miR-588 mimic or mimic NC. The luciferase activity was gauged. This experiment was performed with 3 independent biological repeats × 3 technique repeats. *P < 0.05. **C** RIP assay was applied to verify the binding between circECE1 and miR-588. Lysates of OS cells were incubated with anti-Ago2 or IgG antibody and agarose beads. Beads were harvested, and RNA was processed by qRT-PCR to quantify the levels of circECE1 and miR-588. The assay was performed with 3 independent biological repeats × 3 technique repeats. *P < 0.05. **D** QRT-PCR analysis was adopted for the expression of miR-588 in OS tissues, with U6 as a reference gene. This experiment was performed with 111 independent biological repeats. *P < 0.05. **E** Linear relationship between circECE1 and miR-588 was measured in 111 cases OS samples with Pearson’s correlation analysis. *P* < 0.001. **F** The expression of miR-588 in OS cells was determined through qRT-PCR, with U6 as a reference gene. The assay was performed with 3 independent biological repeats × 3 technique repeats. *P < 0.05

To confirm potential miRNAs associated with circECE1 in OS cells, we used Starbase 3.0 to predict the targeted miRNAs and found that miR-588 bound to circECE1 (Fig. 3A). Subsequently, dual-luciferase reporter assay was performed to prove the interaction between circECE1 and miR-588. We found that miR-588 mimic greatly inhibited the luciferase activity of circECE1 wild type reporter (circECE1 wt), but not the reporter vector (circECE1 mut) containing the mutated pairing sites (Fig. 3B). Meanwhile, RIP results verified that circECE1 and miR-588 were enriched in Ago2 immunoprecipitation, suggesting the association between circECE1 and miR-588 (Fig. 3C). Additionally, in OS tissues, miR-588 expression was reduced (Fig. 3D) and negatively correlated with circECE1 expression (Fig. 3E). MiR-588 expression was also reduced in OS cells (MG63 and U2OS) (Fig. 3F). Collectively, circECE1 targeted miR-588 by sponging miR-588.

### MiR-588 inhibitor partially reverses the effects of circECE1 knockdown in OS cells

**Fig. 4:**
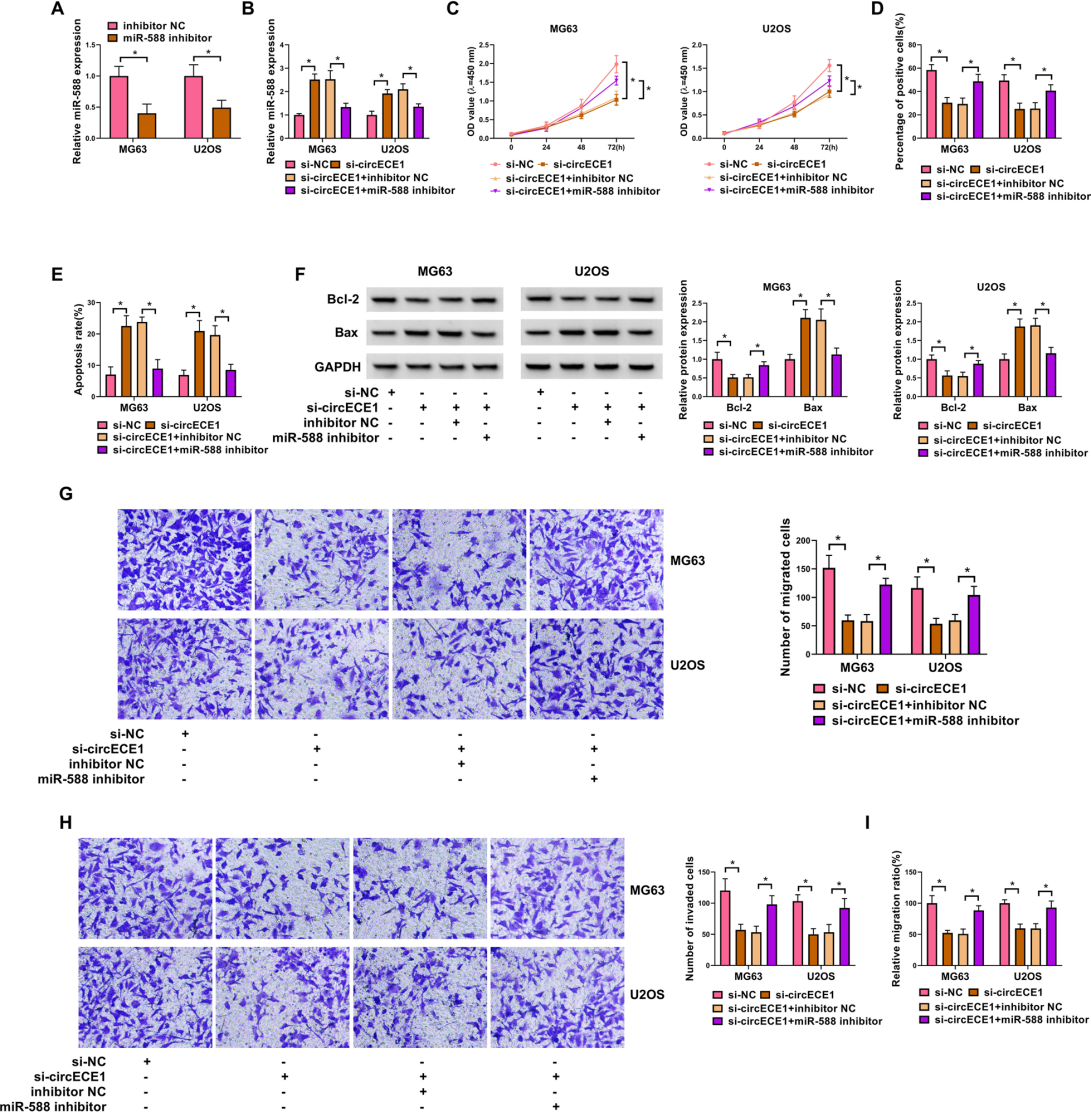
The involvement of miR-588 for circECE1 regulation in OS cells. **A** The expression of miR-588 in OS cells transfected inhibitor NC or miR-588 inhibitor was detected by qRT-PCR, with U6 as a reference gene. (**B**–**I**) MG63 and U2OS OS cells were transfected with si-NC, si-circECE1, si-circECE1 + inhibitor NC or si-circECE1 + miR-588 inhibitor. **B** The expression of miR-588 was detected by qRT-PCR in OS cells transfected with si-NC, si-circECE1, si-circECE1 + inhibitor NC or inhibitor NC + miR-588 inhibitor, with U6 as a reference gene. **C** CCK-8 assay was performed to evaluate OS cell viability. **D** EdU assay indicated OS cell proliferation. **E** OS cell apoptosis was observed through FCM after the above four transfections. **F** Bcl-2 and Bax protein levels were analyzed via western blot, with GAPDH as a loading control. (G and H) The transwell assay assessed the cell migration and invasion. **I** OS cell migration ratio was explored with wound-healing assay after the above four transfections. These experiments **A**, **B**, **C**, **D**, **E**, **F**, **G** and **H** were performed with 3 independent biological repeats × 3 technique repeats. *P < 0.05

MiR-588 expression was clearly silenced in OS cells transfected with miR-588 inhibitor (Fig. 4A). Furthermore, miR-588 expression was enhanced by si-circECE1 introduction, and the effect was partly reversed by co-transfection with miR-588 inhibitor (Fig. 4B). Cell viability was further assessed using CCK-8 assay. CircECE1 knockdown could abate OS cell viability, while the repression was partly recovered by co-transfection with miR-588 inhibitor (Fig. 4C). As revealed in Fig. 4D, circECE1 knockdown inhibited OS cell proliferation, and miR-588 inhibitor abolished this inhibitory effect. Then, we revealed that miR-588 inhibitor reversed the promoting effect of circECE1 knockdown on the apoptosis rate of OS cells (Fig. 4E). In addition, western blot results revealed that miR-588 inhibitor abrogated the impact of circECE1 silencing on the protein levels of Bcl-2 and Bax in OS cells (Fig. 4F). Moreover, miR-588 inhibitor recuperated the inhibitory effects of circECE1 silencing on migration (Fig. 4G) and invasion (Fig. 4H). Similarly, the migration ratio of OS cells (MG63 and U2OS) was notably curbed by si-circECE1, and this effect was weakened by the addition of miR-588 inhibitor (Fig. 4I). To summarize, circECE1 knockdown constrained OS cell malignant behaviors by binding with miR-588.

### RAB3D is a miR-588 target in OS cells

**Fig. 5:**
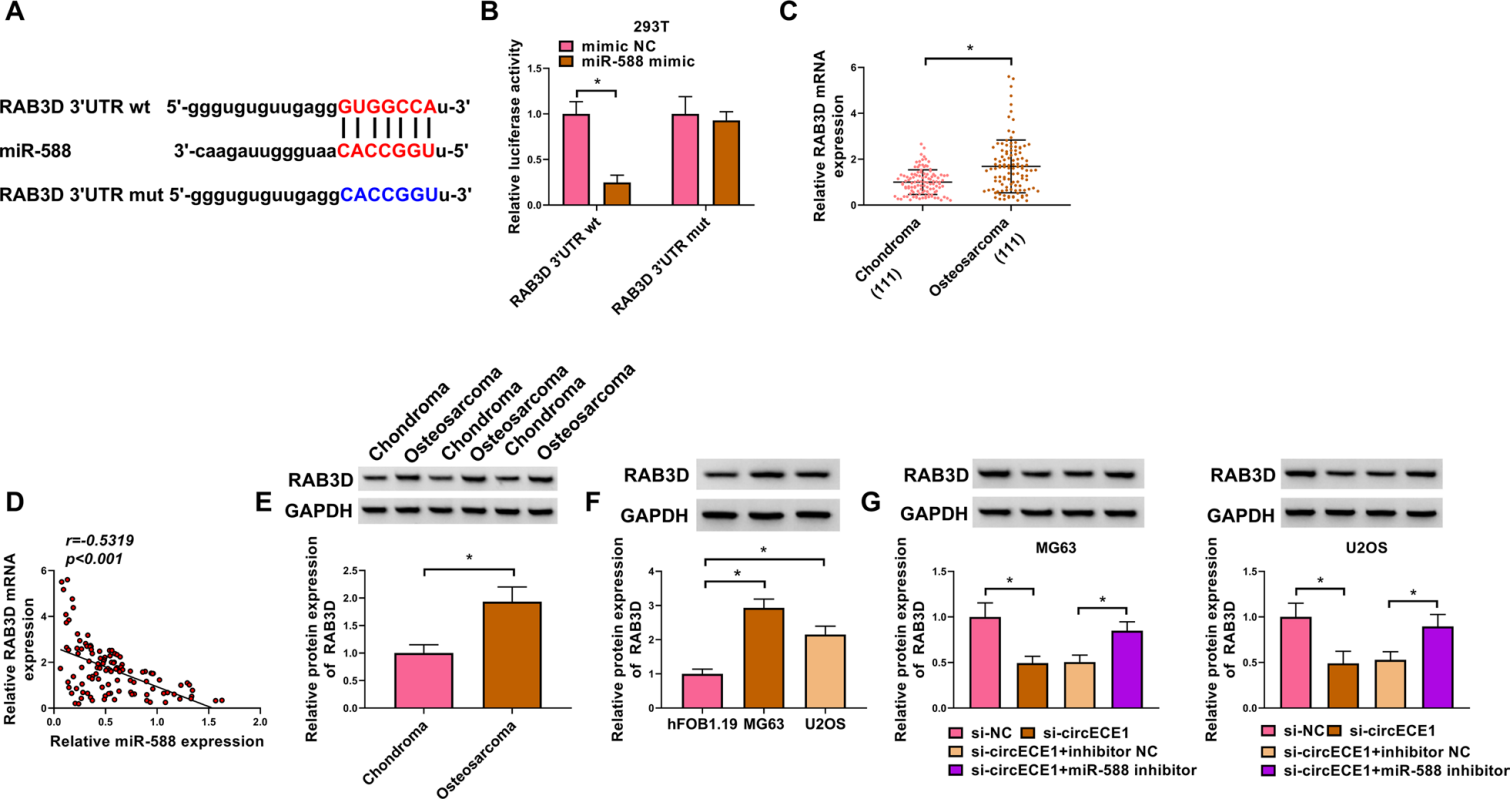
MiR-588 targets RAB3D in OS cells. **A** Starbase 3.0 estimated this binding between miR-588 and RAB3D. **B** Dual-luciferase reporter assay was executed to probe the connection between miR-588 and RAB3D. 293 T cells were transfected with RAB3D 3'UTR wt or RAB3D 3'UTR mut and miR-588 mimic or mimic NC. This experiment was performed with 3 independent biological repeats × 3 technique repeats. *P < 0.05. **C** RAB3D mRNA expression in OS tissues was measured by qRT-PCR, with GAPDH as a reference gene. This experiment was performed with 111 independent biological repeats. *P < 0.05. **D** Pearson’s correlation analysis was applied to discover the relationship between miR-588 and RAB3D in 111 cases OS samples. *P* < 0.001. **E** and **F** RAB3D protein expression was tested by western blot in OS tissues and cells, with GAPDH as a loading control. This experiment was performed with 3 independent biological repeats × 3 technique repeats. *P < 0.05. **G** RAB3D protein expression in OS cells with different transfections (si-NC, si-circECE1, si-circECE1 + inhibitor NC or si-circECE1 + miR-588 inhibitor) was determined by western blot, with GAPDH as a loading control. This experiment was performed with 3 independent biological repeats × 3 technique repeats. *P < 0.05

We predicted that RAB3D was a potential target of miR-588 in OS cells through Starbase 3.0 (Fig. 5A). Next, we proved that miR-588 mimic greatly suppressed luciferase activity of RAB3D 3’UTR wt, but not RAB3D 3’UTR mut, via dual-luciferase reporter assay (Fig. 5B). Moreover, in OS tissues, RAB3D expression was sharply augmented (Fig. 5C) and negatively correlated with miR-588 expression (Fig. 5D). We also found that RAB3D protein expression was significantly upregulated in OS tissues and cells (Fig. 5E and F). CircECE1 silencing diminished RAB3D protein level, and this influence could be relieved by miR-588 inhibitor in OS cells (Fig. 5G). These results demonstrated that miR-588 directly targeted RAB3D.

### RAB3D overexpression partially counteracts the function of miR-588 mimic in OS cells

**Fig. 6:**
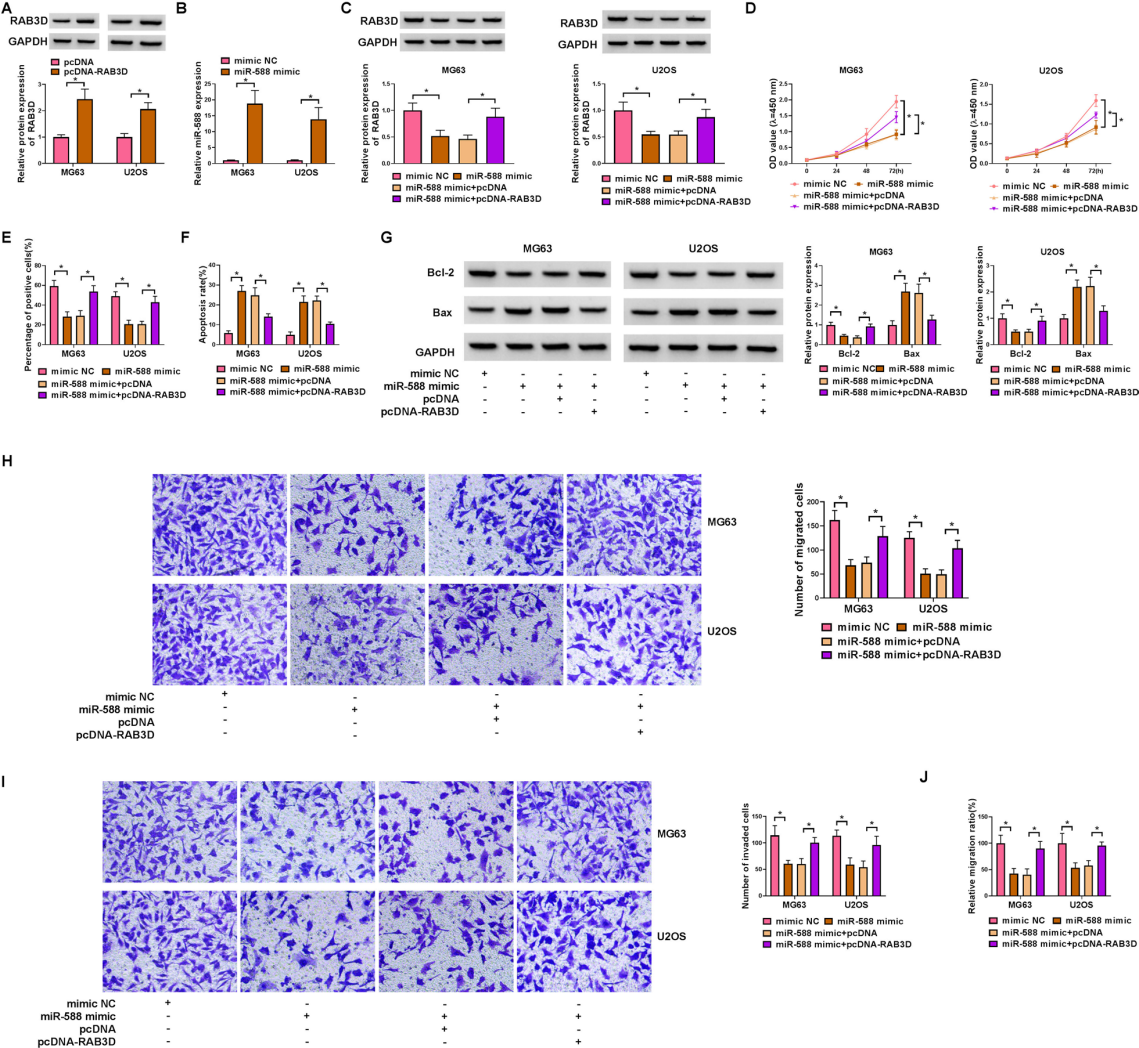
The recovery functions of RAB3D against miR-588 in OS cells. **A** RAB3D protein expression was detected by western blot in OS cells transfected with RAB3D plasmid or pcDNA control, with GAPDH as a loading control. **B** MiR-588 expression was detected by qRT-PCR in OS cells transfected with miR-588 mimic or mimic NC, with U6 as a reference gene. **C**–**J** OS cells were transfected with mimic NC, miR-588 mimic, miR-588 mimic + pcDNA or miR-588 mimic + pcDNA-RAB3D. **C** RAB3D protein expression was conducted by western blot in OS cells transfected with mimic NC, miR-588 mimic, miR-588 mimic + pcDNA or miR-588 mimic + pcDNA-RAB3D, with GAPDH as a loading control. **D** OS cell viability was evaluated by CCK-8 assay. **E** OS cell proliferation was measured by EdU assay. **F** The apoptosis rate was detected in OS cells by FCM. **G** Bcl-2 and Bax protein levels in OS cells were texted by western blot, with GAPDH as a loading control. **H** and **I** Transwell assay was utilized to assess the migration and invasion of transfected OS cells. **J** The migration ratio of OS cells was observed through wound-healing assay. These experiments **A**, **B**, **C**, **D**, **E**, **F**, **G**, **H** and **I** were performed with 3 independent biological repeats × 3 technique repeats. *P < 0.05

RAB3D overexpression plasmid or miR-588 mimic were transfected into OS cells. RAB3D expression was up-regulated by introduction of RAB3D overexpression plasmid (Fig. 6A), and miR-588 expression was elevated by miR-588 mimic (Fig. 6B). To elucidate the effects of miR-588 and RAB3D in OS cells, miR-588 mimic and RAB3D overexpression plasmid were co-transfected into OS cells. RAB3D expression was repressed by miR-588 mimic, and the effect was abrogated by co-transfection with RAB3D overexpression vector (Fig. 6C). Functional experiments showed that overexpression of RAB3D abolished the inhibitory functions of miR-588 mimic in OS cell viability (Fig. 6D) and proliferation (Fig. 6E). Overexpression of RAB3D also abolished the promoting effect of miR-588 mimic on OS cell apoptosis (Fig. 6F). Western blot was used to detect Bcl-2 and Bax protein levels, and the results indicated that overexpression of RAB3D abated the influence of miR-588 mimic on Bcl-2 and Bax protein expression (Fig. 6G). Moreover, RAB3D overexpression abrogated the inhibitory effects of miR-588 mimic on cell migration and invasion (Fig. 6H–J).

### CircECE1 promotes tumor growth in OS

**Fig. 7:**
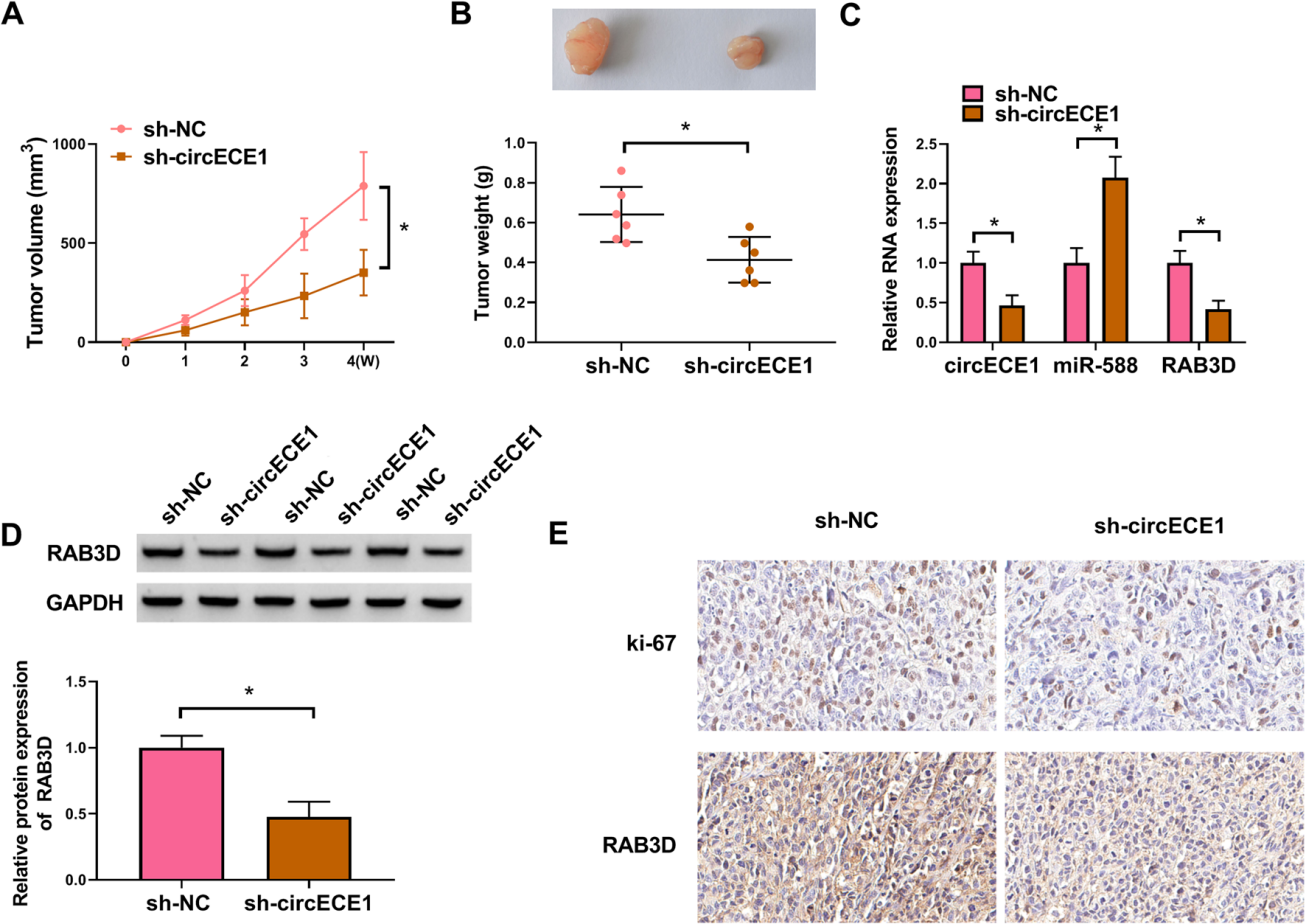
CircECE1 knockdown restricts tumor growth. **A**–**E** MG63 cells stably transfected with sh-NC or sh-circECE1 lentivirus were subcutaneously injected into nude mice (6 mice/group). Four weeks later, tumors were removed from the mice. **A** Tumor volume was observed in sh-circECE1 and sh-NC group. **B** Tumor weight was measured after 4 weeks in sh-circECE1 and sh-NC group. **C** RNA expression of circECE1, miR-588 and RAB3D in sh-circECE1 and sh-NC group was detected by qRT-PCR. **D** Western blot was performed to assess RAB3D protein level in sh-circECE1 and sh-NC group, with GAPDH as a loading control. **E** IHC analysis was implemented to examine the expression of Ki-67 and RAB3D in sh-circECE1 and sh-NC group. These experiments **A**, **B**, **C**, **D** and **E** were performed with 6 independent biological repeats. *P < 0.05

We further explored the function of circECE1 silencing in vivo by using OS cells stably transfected with sh-circECE1 or sh-NC lentivirus. After 4 weeks of cell injection, tumor volume (Fig. 7A) and weight (Fig. 7B) were obviously declined in the sh-circECE1 group compared to the sh-NC group. Meanwhile, compared with the sh-NC group, circECE1 and RAB3D mRNA levels were notably down-regulated while miR-588 expression was up-regulated (Fig. 7C) in the sh-circECE1 group. Furthermore, RAB3D protein level was decreased in the sh-circECE1 group (Fig. 7D). Finally, the cells stained for Ki-67 and RAB3D in tumor tissues of the sh-circECE1 group were fewer than those in sh-NC group, as measured by IHC assay (Fig. 7E). Collectively, circECE1 silencing could lead to the repression of tumor growth in vivo.

## Discussion

Multiple factors are associated with the development of OS, and the disease is highly variable and very difficult to treat, leading to poor prognosis [23]. Therefore, innovative therapies against OS are urgently required.

Many studies have shown that circRNAs actively participate in the progression of many cancers by controlling importantly cellular processes such as proliferation, migration and invasion [24]. For instance, circ_0028171 silencing mediates OS progression via competitively binding to miR-218-5p [25]. Circ_0005909 promotes cell growth and drug resistance through miRNA-338-3p/SOX4 axis in non-small cell lung cancer [26]. Yue et al*.* disclosed that circ_0004104 knockdown affects the malignant phenotypes of gastric cancer (GC) cells, suggesting that circ_0004104 might be a GC biomarker for diagnosis and treatment [27]. Yang et al*.* exhibited that the hsa_circ_0026123/miR-124-3p/EZH2 axis could play critical role in ovarian cancer [28]. Here, we illustrated that circECE1 is markedly up-regulated in OS, consistent with a previous result [11]. Furthermore, circECE1 knockdown by a specific siRNA curbed tumor growth in vivo, attenuated OS cell viability, proliferation, migration, and invasion and promoted OS cell apoptosis in vitro, suggesting that the siRNA targeting circECE1 may be a therapeutic method for OS. Similarly, siRNAs targeting other related genes have been shown to have potential values for tendon repair and rheumatoid arthritis management [29, 30].

MiRNAs play an indelible role in many OS cell malignant behaviors [31]. For example, miR-95 deficiency causes decreased proliferation and increased apoptosis in OS cells [32]. Furthermore, miR-421 is able to control MCPIP1 expression through negative regulation, thus promoting OS tumorigenicity in vivo [33]. Our study confirmed that miR-588 is down-regulated in OS cells, consistent with recent literature reports [19, 34]. MiR-588 downregulation is reported to be tightly correlated with Musculoskeletal Tumor Society (MSTS) staging in OS patients [19]. MiR-588 downregulation also dramatically enhances the proliferation, migration and invasive abilities of OS cells [19]. Liu et al*.* discovered that miR-588 inhibitor distinctly expedites the invasive and migratory capacity of OS cells in vitro [34]. In the present work, we proved that circECE1 serves as a sponge for miR-588 and negatively regulated miR-588. MiR-588 inhibitor overturned these effects of circECE1 knockdown on OS cell viability, proliferative capacity, metastatic capacity, and apoptosis.

In recent years, the importance of RAB3D in OS has become increasingly clear. Wang et al*.* suggested that RAB3D can be negatively regulated by miR-627-3p, thereby regulating OS development [21]. Cao et al.displayed that RAB3D can facilitate OS cell proliferation, migration, and invasion [22]. In our research, we elaborated the targeting of RAB3D by miR-588. Also, circECE1 regulates RAB3D expression by targeting miR-588. More importantly, RAB3D overexpression reverses the alterations of OS cell malignant phenotypes induced by miR-588 mimic.

Based on these findings, the circECE1 siRNA may represent a potential anti-OS agent that does not act as a repressor of OS cell growth and metastasis but induces cell apoptosis. We envision that the circECE1 siRNA is an important point for development of molecularly targeted therapies against OS. In addition, the efficacy and safety of the circECE1 siRNA in various animal models should be assessed. Although our study has highlighted the implication of the circECE1/miR-588/RAB3D regulatory axis in OS, some limitations still exist in the current research. The downstream signaling pathways of RAB3D remain to be determined. The role of circECE1/miR-588/RAB3D axis in regulating tumor metastasis in vivo needs to be further analyzed using various experimental models.

**Fig. 8:**
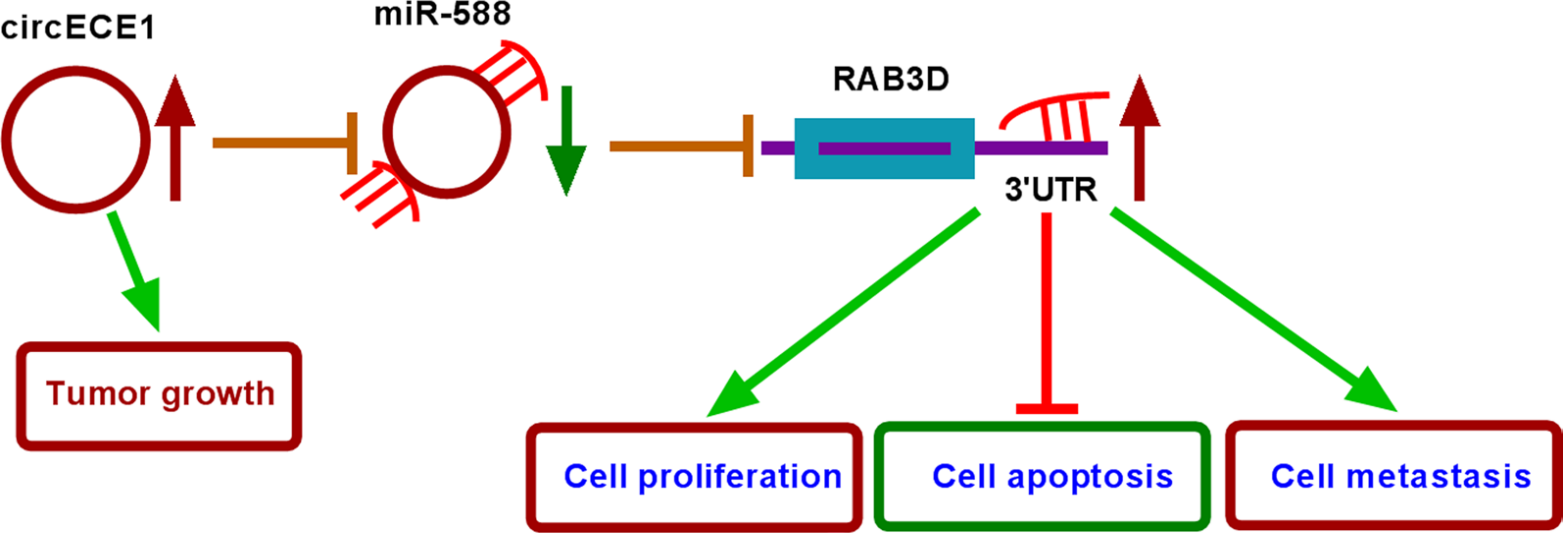
The schematic diagram shows the role of circECE1/miR-588/RAB3D axis in OS progression

Taken together, we demonstrated, for the first time, the circECE1/miR-588/RAB3D regulatory axis in OS (Fig. 8). CircECE1 inhibition may be a novel therapeutic strategy for OS.

## Data Availability

The datasets used and analyzed during the current study are available from the corresponding author on reasonable request.
